# Faster cytotoxicity with age: Increased perforin and granzyme levels in cytotoxic CD8 ^+^ T cells boost cancer cell elimination

**DOI:** 10.1111/acel.13668

**Published:** 2022-07-11

**Authors:** Dorina Zöphel, Adrian Angenendt, Lea Kaschek, Keerthana Ravichandran, Chantal Hof, Sandra Janku, Markus Hoth, Annette Lis

**Affiliations:** ^1^ Biophysics, Center for Integrative Physiology and Molecular Medicine School of Medicine, Saarland University Homburg Germany; ^2^ Cellular Neurophysiology, Center for Integrative Physiology and Molecular Medicine, School of Medicine Saarland University Homburg Germany

**Keywords:** aging, CD8^+^ T cells, CTL, cytotoxicity, granzyme, immunosenescence, perforin, tumor immunology

## Abstract

A variety of intrinsic and extrinsic factors contribute to the altered efficiency of CTLs in elderly organisms. In particular, the efficacy of antiviral CD8^+^ T cells responses in the elderly has come back into focus since the COVID‐19 pandemic outbreak. However, the exact molecular mechanisms leading to alterations in T cell function and the origin of the observed impairments have not been fully explored. Therefore, we investigated whether intrinsic changes affect the cytotoxic ability of CD8^+^ T cells in aging. We focused on the different subpopulations and time‐resolved quantification of cytotoxicity during tumor cell elimination. We report a surprising result: Killing kinetics of CD8^+^ T cells from elderly mice are much faster than those of CD8^+^ T cells from adult mice. This is true not only in the total CD8^+^ T cell population but also for their effector (T_EM_) and central memory (T_CM_) T cell subpopulations. TIRF experiments reveal that CD8^+^ T cells from elderly mice possess comparable numbers of fusion events per cell, but significantly increased numbers of cells with granule fusion. Analysis of the cytotoxic granule (CG) content shows significantly increased perforin and granzyme levels and turns CD8^+^ T cells of elderly mice into very efficient killers. This highlights the importance of distinguishing between cell‐intrinsic alterations and microenvironmental changes in elderly individuals. Our results also stress the importance of analyzing the dynamics of CTL cytotoxicity against cancer cells because, with a simple endpoint lysis analysis, cytotoxic differences could have easily been overlooked.

## Graphical abstract

Immunosenescence is associated with decreased T cell numbers and impaired functionality of cytotoxic T lymphocytes (CTL). Surprisingly, we find that, despite immunosenescence, CTL efficiency against cancer cells increases with age, if similar CTL numbers or individual cells are compared. Elevated perforin and granzyme levels turn CTLs of elderly mice into ultimate killers, highlighting the importance of distinguishing cell intrinsic alterations from microenvironmental changes in elderly individuals.

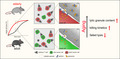

## 1 INTRODUCTION

Aging is associated with reduced functionality and altered distribution of the immune cells from innate and adaptive immunity in human and mice (Nikolich‐Zugich, 2018; Pinti et al., 2016; Weiskopf et al., 2009). Especially, T cells' behavior undergoes crucial alterations during aging, resulting in susceptibility to infectious diseases (Goronzy & Weyand, 2017; Nikolich‐Zugich et al., 2012) and a higher risk of cancer (Foster et al., 2011; Pawelec, 2017). Age‐related changes have been reported in almost all subpopulations of the CD4^+^ and CD8^+^ T cell compartments, but with different phenotypic characteristics (Minato et al., 2020). A major factor contributing to age‐related defects in immunological responses is the progressive deterioration of naïve CD8^+^ T cell function (Fagnoni et al., 2000), including reduced expansion upon activation (Decman et al., 2010; Jiang et al., 2013), altered cytokine production (Engwerda et al., 1996; Mirza et al., 2011), and production of altered memory T cell population (Goronzy & Weyand, 2017).

Furthermore, intrinsic and extrinsic factors influence the decreased CD8^+^ T cell response with age (Decman et al., 2010; Decman et al., 2012; Linton et al., 2005; Yager et al., 2008; Zöphel et al., 2020), but the exact mechanisms are still not fully understood.

Upon primary infection, naïve CD8^+^ T cells (T_N_) are activated by antigen‐presenting cells, clonally expand, and differentiate into multiple subsets, including memory CD8^+^ T cells, namely, central memory T cells (T_CM_) and effector memory T cells (T_EM_) (Sallusto et al., 1999; Williams & Bevan, 2007).

An additional level of complexity is the extensive heterogeneity and plasticity of these subpopulations, implying that different CD8^+^ T cell subsets, endowed with specific phenotypic and functional features, may be involved and play distinct roles in the elimination of target cells.

Many expectations rest on these immunocompetent cells, including recognizing, targeting and excellent cytotoxic activity against tumor cells, especially within cancer immunotherapy. Critical for the success of adoptive T cell therapy of patients with infection or tumor is the ability to expand specific T cells harvested from patient's blood without losing functional capabilities. Therefore, the use of tumor‐reactive CD8^+^ cytotoxic T lymphocytes (CTL) requires efficient in vitro approaches allowing not only the expansion of CTLs to large numbers but improvement and preservation of cytotoxicity to achieve a significant tumor removal or reduction. The pool of lymphocytes used for adoptive immunotherapy can be derived from any of the CD8^+^ T cell subsets, including T_N_, T_CM_ and T_EM_ that differ in phenotype, homing, and function (Contreras et al., 2018; Sommermeyer et al., 2016). It has been shown that effector cells derived from T_CM_ rather than T_EM_ possess increased potential to survive and establish immunologic memory after infusion (Klebanoff et al., 2005). The identification and characterization of functionally relevant subsets with excellent cytotoxic ability can be used to predict the potential efficacy of an efficient immune response. Initial successes have already been achieved for adaptive immunotherapy with T cells from young organisms (Rosenberg et al., 2011). However, during aging, we are facing an even more complex and altered scenario. The elderly T cell subsets differ not only from those of a healthy organism per se but also from the adult.

Since aging results in a continuous remodeling and adaptation of the immune system and affect CD8^+^ T cells, more profound information is necessary to improve their impaired function. Despite the evidence that the immune system of the aged is significantly altered, there is increasing data suggesting that particularly CD8^+^ T cells can remain functionally intact and show even enhanced cytotoxicity (Saxena & Adler, 1999; Saxena et al., 1988). Functional properties of each expanded T cell subset may pave the way for a more detailed evaluation of the effects of aging‐related changes on the subset contribution to in vitro‐expanded T cells. To distinguish the potential effects of the aged host from the intrinsic quality of memory CD8^+^ T cells from aged mice, we focus on their potential cytotoxic activity against tumor cell lines.

## 2 RESULTS

### 2.1 CD8 ^+^ T cells from elderly mice show distinct kinetics against different tumor cell lines

CD8^+^ T cells' essential function is to detect and eliminate potentially harmful cells by inducing apoptosis. However, little is known about the killing kinetics of different CD8^+^ T cell subtypes, especially during aging.

**FIGURE 1:**
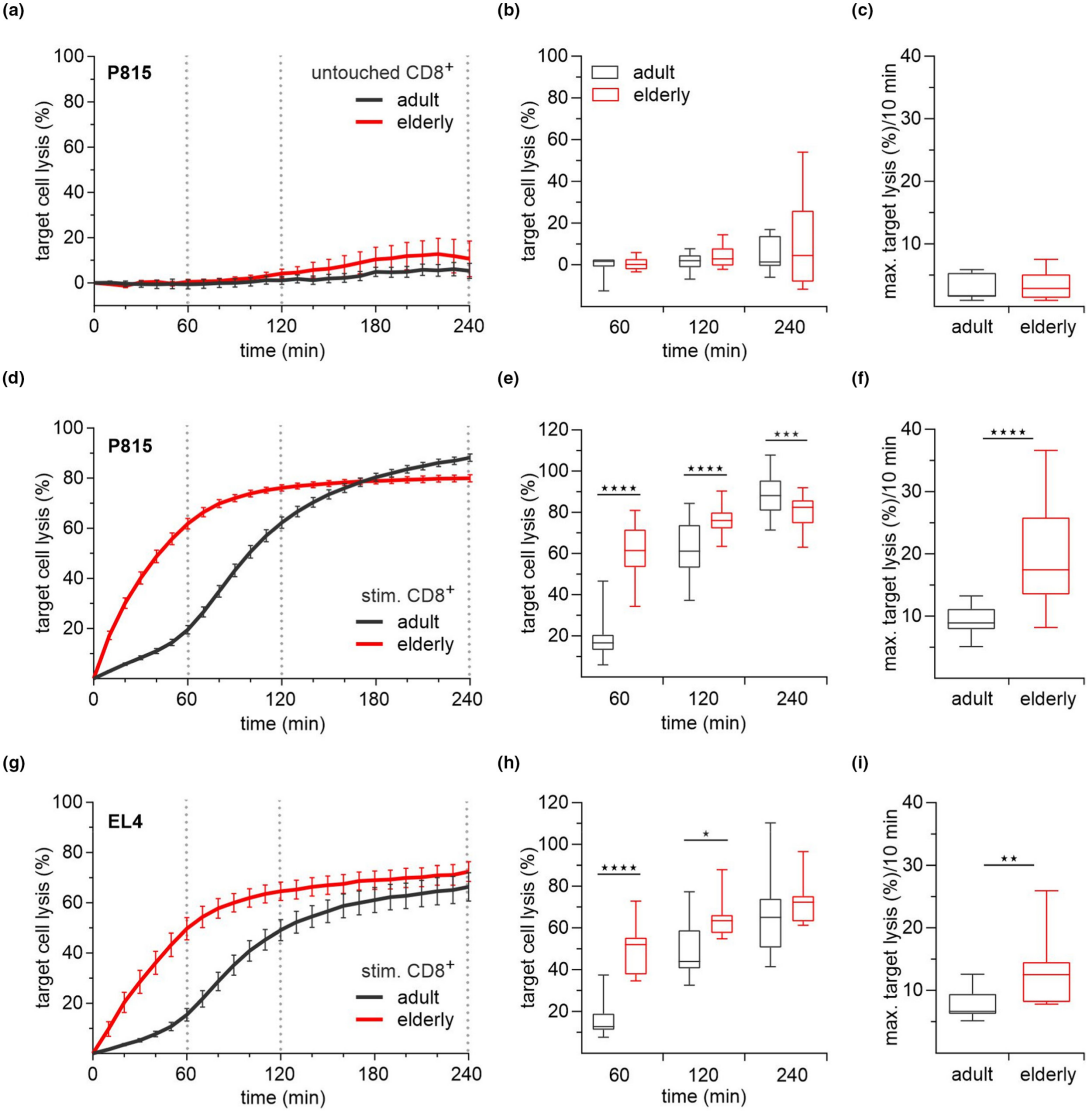
Faster killing kinetics of CD8^+^ T cells from elderly mice. Time‐resolved killing assays with untouched (a) (*n* = 7–8) and stimulated (d, g) CD8^+^ T cells from adult and elderly mice. P815 (*n* = 31–35) and EL4 (*n* = 8–11) tumor cell lines were used as target cells in an effector‐to‐target ratio of 20:1. Box plots represent the average target cell lysis after 60, 120, and 240 min (b, e, h) and the maximum target lysis per 10 min (c, f, i) as a measure of the kinetics. Data are presented as mean ± SEM

First, we tested the cytotoxic ability of untouched CD8^+^ T cells to kill their target cells using a calcein‐based time‐resolved killing assay (Kummerow et al., 2014). We assayed cytotoxic activity using P815 cells as target cells loaded with the fluorescent dye calcein‐AM to directly monitor the loss of fluorescence during target cell lysis. However, the untouched CD8^+^ T cells did not acquire any cytotoxic activity a few hours after isolation from the spleen of young and elderly mice (Figure 1a–c). After 3 days of polyclonal stimulation with CD3/CD28 beads, pronounced lysis of the P815 cells was detected for both age groups (Figure 1d) with higher end point lysis for CD8^+^ T cells from young mice (Figure 1d,e). However, the activated CD8^+^ T cells of elderly mice display significantly faster kinetics to lyse the target cells (Figure 1d). The red curve representing the cells of the elderly mouse showed a steeper, hyperbolic increase in contrast to the sigmoid course of the black curve of the CD8^+^ T cells of adult mice (Figure 1d). This continuous significant increase in the cytotoxic activity for CD8^+^ T cells from elderly mice was compensated by CD8^+^ T cells from adult mice only after 180 min into the assay (Figure 1d,e). Furthermore, we analyzed the maximum target lysis rate as a sensitive parameter for the cells' characterization of lysis capacity. The quantification of the maximum target lysis rate, as the maximum slope of the curves in a time interval of 10 min, resulted in a significantly faster target cell lysis of the CD8^+^ T cells of elderly mice, almost twice as fast as compared to the CD8^+^ T cells of the adult mice (Figure 1f). To rule out that these effects only apply to the P815 cells, we confirmed the kinetic by using an additional lymphoma cell line EL4 (Figure 1g). The cytotoxicity assay results with EL4 cells as the target cell line (Figure 1g–i) were comparable to P815 target cells (Figure 1d–f). The CD8^+^ T cells of elderly mice showed significant differences, including the faster kinetics (Figure 1g), maximum target cell lysis (Figure 1i) but with similar endpoint lysis (Figure 1g,h). Since the effector‐to‐target cell ratio determines the cytotoxic activity and might impact the cell lysis kinetics, we assayed the target cells at various effector‐to‐target cell ratios (Figure S1). The cytotoxic activity's overall magnitude decreased with the decreasing ratios as expected; however, the different kinetic courses between adult and elderly mice were conserved (Figure S1a,d).

Moreover, to exclude whether differences in the cell lysis kinetics resulted from differential cell proliferation, we labeled the cells with CFSE and tracked their proliferative capacity through early rounds of cell division (every 24 h) after polyclonal stimulation (Figure S2). At 48 h, a significantly greater proportion of CD8^+^ T cells of adult mice were compared to the elderly, in divisions 3 and 4 (Figure S2a,b), reflecting their greater proliferative capacity as previously described (Decman et al., 2010; Smithey et al., 2011). Besides the increased proliferation, the viability of CD8^+^ T cells of adult mice was significantly better compared to the elderly (Figure S2c) at the time point of performing the killing assay (after 72 h). Collectively, these data show that CD8^+^ T cells of elderly mice have a faster cytotoxic activity than their younger counterparts despite their reduced proliferative capacity and viability. Furthermore, dysregulations of IL‐2 concentrations (in vivo and in vitro) might have a dramatic impact on the generation of effector and memory T cells, and IL‐2 is essential for their proliferation and survival (Kalia & Sarkar, 2018; Ross & Cantrell, 2018). To test the influence of IL‐2 on the activation of CD8^+^ T cells from adult and elderly mice and a possible indirect impact on the killing kinetics, we stimulated them in the presence of different IL‐2 concentrations and tested for cytotoxic ability (Figure S3). IL‐2 concentrations significantly affected endpoint lysis of target cells in CD8^+^ T cells from adult mice (Figure S3a–c). Surprisingly, the CD8^+^ T cells from elderly mice hardly responded to changes in IL‐2 concentration during stimulation (Figure S3d–f). Although it appears that IL‐2 has an impact on end‐point lysis in the younger, kinetics remained unaffected in both cohorts.

### 2.2 CD8 ^+^ T cell subtype distribution is not decisive for the faster kinetics

**FIGURE 2:**
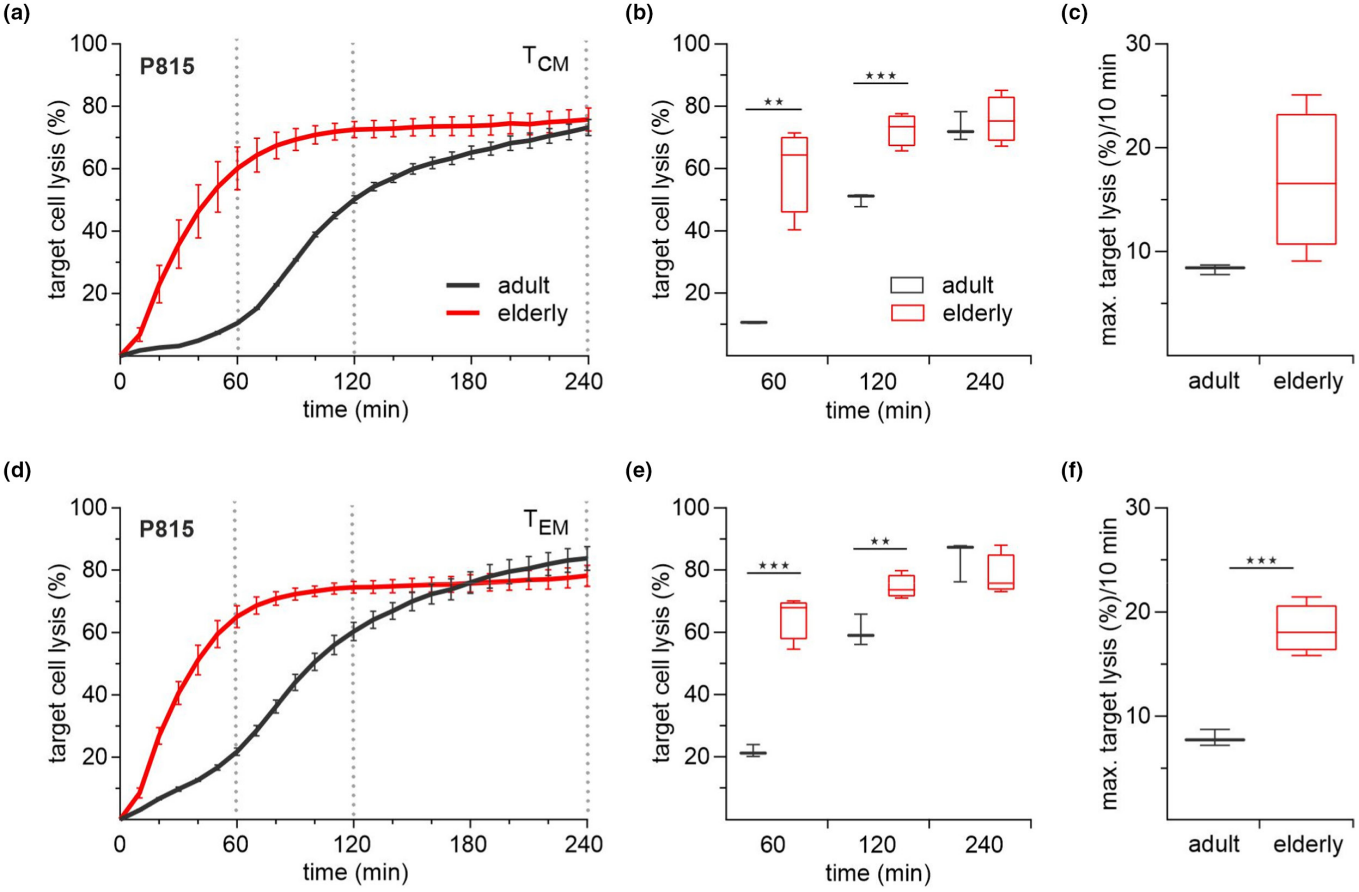
Central and effector memory CD8^+^ T cells from elderly mice show fast killing kinetics. Time‐resolved killing assays with stimulated and sorted CD8^+^ T cell subtypes T_CM_ (a) and T_EM_ (d) from adult (*n* = 3) and elderly (*n* = 4) mice and P815 as target cells in an effector‐to‐target ratio of 20:1. Box plots represent the average target cell lysis after 60, 120, and 240 min (b, e) and the maximum target lysis per 10 min (c, f). Data are presented as mean ± SEM

A shift in the distribution of T cell subtypes to less naïve and more memory cells is considered a marker of immunosenescence. CD8^+^ T cells of adult mice mainly consist of naϊve cells and shift primarily to central (T_CM_) and secondarily to effector memory (T_EM_) cells. In contrast, the CD8^+^ T cell pool of elderly mice mostly consists of T_CM_ and shifts to more T_EM_ with T cell activation (Angenendt et al., 2020). Additionally, several publications reported higher perforin expression levels with increased cytotoxic activity for T_EM_ than the T_CM_ cell subpopulation (Sallusto et al., 1999; Willinger et al., 2005). Thus, one would expect that the faster kinetics of cytotoxicity in elderly mice is mainly based on the age‐related differences in the CD8^+^ T cell subtype distribution. To test this hypothesis, we performed cytotoxicity assays with stimulated and sorted T_CM_ and T_EM_ subpopulations (Figure 2). Data analysis revealed quite similar kinetics for both T_CM_ (Figure 2a,b) and T_EM_ (Figure 2d,e) subpopulations from elderly mice, with T_EM_ cells from adult mice displaying slightly faster kinetics than T_CM_ (Figure 2a,b,d,e). The quantification of the kinetics as quantified by the maximum lysis rate per 10‐min interval reveals a two‐fold increase for the T_EM_ and T_CM_ of elderly mice compared to adult mice's corresponding subtypes (Figure 2c,f). Since the T_CM_ and T_EM_ subpopulations display almost similar cytotoxic kinetics (Figure 2a,d) within CD8^+^ T cells of the adult and the elderly, their abundance and proportion do not seem to be involved in shaping the faster kinetics of CTLs from elderly mice.

However, within the untouched CD8^+^ T cells from adult mice, especially the naïve T cells make up the largest population (Angenendt et al., 2020) and shift the ratio between the subpopulations significantly. Moreover, the naïve T cell needs a certain stimulation level to become activated via TCR and CD28 co‐stimulatory receptors (Curtsinger et al., 1998). Therefore, one would expect the naïve T cell presence in CD8^+^ T cells from adult mice may influence their cytotoxic competence and ability to a greater degree due to incomplete activation. To test the hypothesis, we isolated the naïve T cells from splenocytes of adult and elderly mice, and after three‐day stimulation, the cytotoxic ability was quantified (Figure S4). Due to the limited number of cells after isolation and stimulation, a 10:1 effector‐to‐target ratio against P815 was investigated. No cytotoxic activity for the polyclonal stimulated CD8^+^ T cells from the naïve T cell population was observed (Figure S4a–c). The flow cytometry analysis revealed a large population of naïve and T_CM_ with a small T_EM_ population for both age groups (Figure S4d,e). The increased abundance of naïve T cells after 3‐day stimulation indicates decreased or time‐delayed activation compared to untouched CD8^+^ T cell stimulation, as already reported (Angenendt et al., 2020). To exclude the influence of incomplete activated CD8^+^ T cells descended from T_N_ subpopulation in adult mice sorted T_CM_/T_EM_ were stimulated, without or in the presence of T_N_ cells in 60:40% ratio for 3 days (Figure S4f,g). The absence or presence of the adult naïve T cells during stimulation did not affect the kinetics in any way (Figure S4h–j). Both killing curves show the typical sigmoidal curve of CD8^+^ T cells isolated from the adult mice (Figure S4h,i), with a slightly reduced cytotoxic activity. This data supports the conclusion that the proportion of naïve T cells from the adult and elderly mouse has only a minimal influence, if any, on the slowed killing kinetics.

Taken together, neither the target‐to‐effector cell ratio, viability, proliferation, IL‐2 concentrations nor the T_EM_ to T_CM_ ratio and the naïve T cells proportion explain the differences in the observed killing kinetics of the CD8^+^ T cells isolated from adult and elderly mice.

### 2.3 CD8 ^+^ T cells from adult mice fail to kill target cells during the first 2 h of cytotoxic activity

**FIGURE 3:**
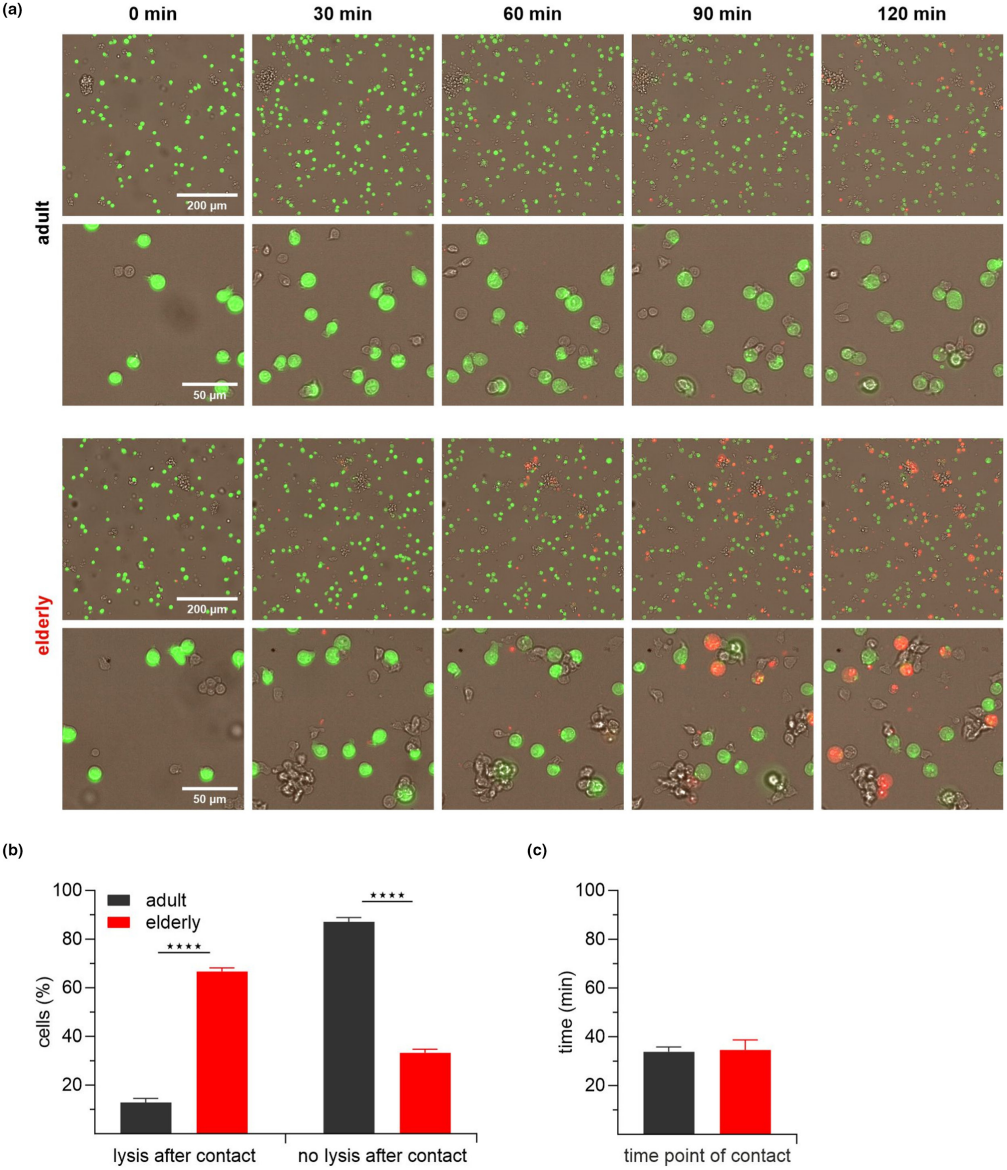
Live‐cell imaging confirms rapid target cell lysis by CD8^+^ T cells from elderly mice. Stimulated CD8^+^ T cells were added to calcein labelled P815 cells. Cells were imaged every 2 min with propidium iodide (PI) in the media. (a) Representative overlays with fluorescence of calcein (green), PI (red) and brightfield (grey) after 0, 30, 60, 90 and 120 min are depicted. (b) Quantitative analysis of target cell lysis after CTL contact. (c) Time points of the first CTL‐target cell contact regardless of the cell fate. Bar graphs show values as mean ± SEM, *n* = 3 mice, each with 60–90 analyzed cells

To verify and visualize the kinetic differences observed with the cytotoxicity assay, we directly monitored single cell killing in parallel using an automated high‐content microscopy system at a 2:1 effector‐to‐target ratio. Since necrosis leads to irreversible cell injury, with loss of plasma membrane integrity and rapid death, we combined two dyes in this set of experiments. We loaded calcein in P815 cells, and propidium iodide was kept in the supernatant as an additional indicator for cell death caused by necrosis. As expected, rapid lysis of target cells by CD8^+^ T cells from elderly mice was observed (Figure 3) already after 30 min with a significantly higher amount of target cell lysed after 120 min (Figure 3a). Interestingly, we observed that most CD8^+^ T cells from adult mice form close cell‐to‐cell contacts but show a lack of or very delayed lysis (Figure 3b). However, the time point of first contact, which is a measure how long the CTLs need to find their first targets, was not significant between both age groups (Figure 3c).

### 2.4 Adult and elderly CD8 ^+^ T cells show similar cytotoxic granule fusion

**FIGURE 4:**
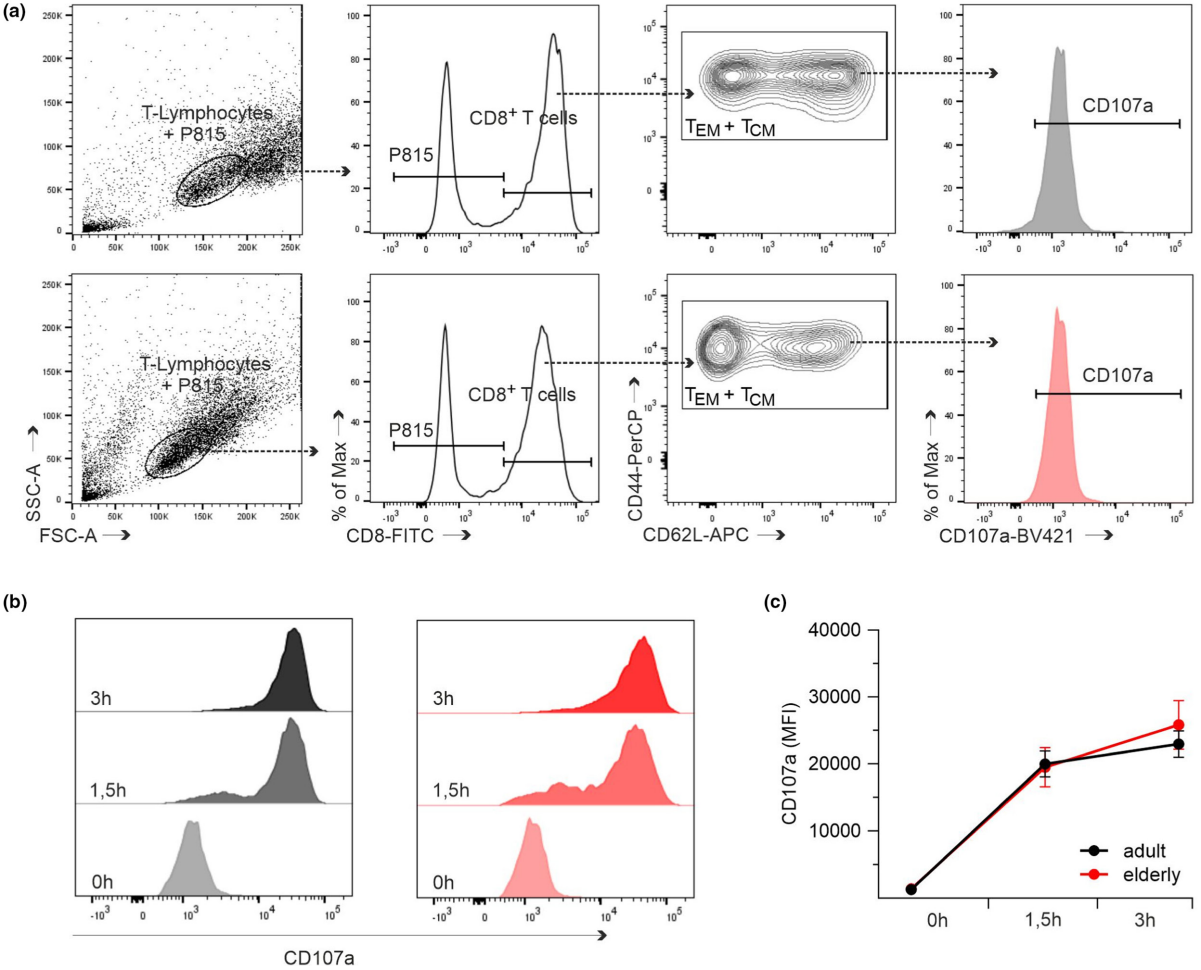
CD8^+^ T cells from adult and elderly mice show similar degranulation ability. Flow cytometry‐based analysis of CD107a (LAMP‐1) mobilization in stimulated CD8^+^ T cells during cytotoxic activity. (a) Representative gating strategy for CD8^+^ T cells from adult (black) and elderly (red) mice immediately after co‐culturing with P815 target cells (effector‐to‐target ratio 1:1). Viable CD8^+^ T cells were defined based on SSC‐A (side scatter area) vs. FSC‐A (forward scatter area) and CD8 surface expression. Expression of CD107a was determined in CD44^+^CD62L^+^ (T_CM_) and CD44^+^CD62L^−^ (T_EM_) cells. (b) Representative histograms of CD107a surface expression in T_CM_ and T_EM_ from adult (black) and elderly (red) mice at different time points after co‐culturing with P815 target cells. (c) Time course of CD107a mobilization analyzed by mean fluorescence intensity (MFI). Data are presented as mean ± SEM, *n* = 6

**FIGURE 5:**
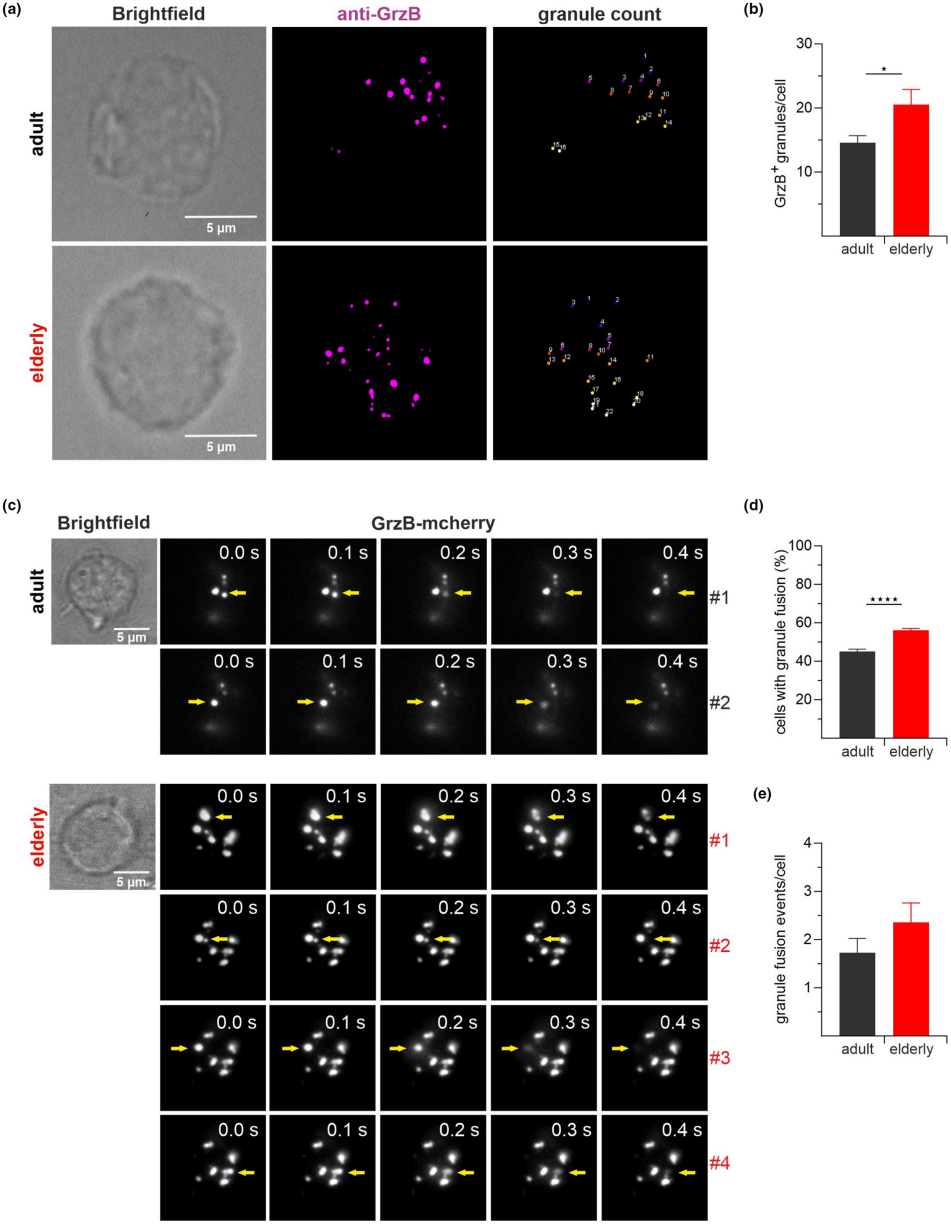
Similar lytic granule events but increased numbers of CTLs with granule fusion in old age. Stimulated CD8^+^ T cells were transfected with granzyme B‐mcherry construct for 12 h and settled on an anti‐CD3 coated coverslip. Representative SIM images (a) and analysis of GrzB^+^ vesicles per cell (b), *n* = 6–7. Representative TIRF images showing the individual fusion events of lytic granules are marked by an arrow (c). The numbers on the right of each image represents the number of fusion events in the particular cell i.e., two fusion events by the CD8^+^ T cell of the adult mouse and 4 fusion events by CD8^+^ T cell from the elderly. Bar graphs show the percentage of CD8^+^ T cells with granule fusion (d), and the average number of granule fusion events per cell (e), *n* = 5. Data are presented as mean ± SEM

As a critical component of cytotoxic granules, granzyme B participates in T cell‐mediated target cell killing. An effective killing depends on the exclusive release of granule content at the immunological synapse and represents an additional parameter to influence CD8^+^ T cells' killing kinetics. During degranulation, cytolytic granules release their contents, and the lysosome‐associated membrane protein‐1 (LAMP‐1, CD107a) is transported to the cell surface and becomes accessible for antibody binding (Betts et al., 2003). The cell surface expression of CD107a indicates recent cytotoxic vesicle degranulation and makes it an attractive marker for assessing the integrity of the granule exocytosis mechanism. Because adult and elderly CD8^+^ T cells show different kinetics in the real time cytotoxicity assay, we wondered if the degranulation of GC granules mirrors the observed kinetics. Besides, this method could provide initial indications of possible defects/alterations in granule fusion. Therefore, we used a flow cytometry‐based degranulation method to measure CD107a (LAMP‐1) mobilization in CD8^+^ T cells. Murine CD8^+^ T cells isolated from adult and elderly mice were co‐cultured with P815 as target cells in 1:1 effector‐to‐target ratio and analyzed at different time points (0, 1.5, and 3 h). The entire population of T_EM_ and T_CM_ (Figure 4a presents the gating strategy) of adults were compared against the elderly (Figure 4). Statistical analysis of CD107a expression (as mean fluorescence intensity, MFI) showed no significant differences in the degranulation at investigated time points (Figure 4b,c). These results indicate that differences between adult or elderly CD8^+^ T cells' ability to induce target cell death is not based on the act of degranulation itself. To receive a more detailed analysis, we performed structured illumination microscopy (SIM) on single cells (Figure 5a,b) and TIRF experiments (Figure 5c–e) by monitoring fusion events of granzyme B^+^ granules and their overall numbers. The analysis of granzyme B‐mcherry positive CTLs from elderly mice revealed significantly increased cell numbers with granule fusion compared to their younger counterparts (Figure 5c,d). The elderly CD8^+^ T cells also show a significance for granzyme B^+^ granules (Figure 5a,b) and more granule fusion events per cell (Figure 5e). Comparing the data from Figures 4 and 5, we conclude that the discrepancy between degranulation and lytic effector function could be related to differences in lytic granule content.

### 2.5 Age‐related increased expression of perforin and granzyme in CD8 ^+^ T cells from elderly mice

Since the possibilities investigated so far may contribute to the altered killing kinetics but certainly do not fully explain it, we decided to focus more closely on the killing process's key components. It has been reported that higher perforin concentrations can lead to the target cell membrane's rupture, causing necrosis of the affected cell.

**FIGURE 6:**
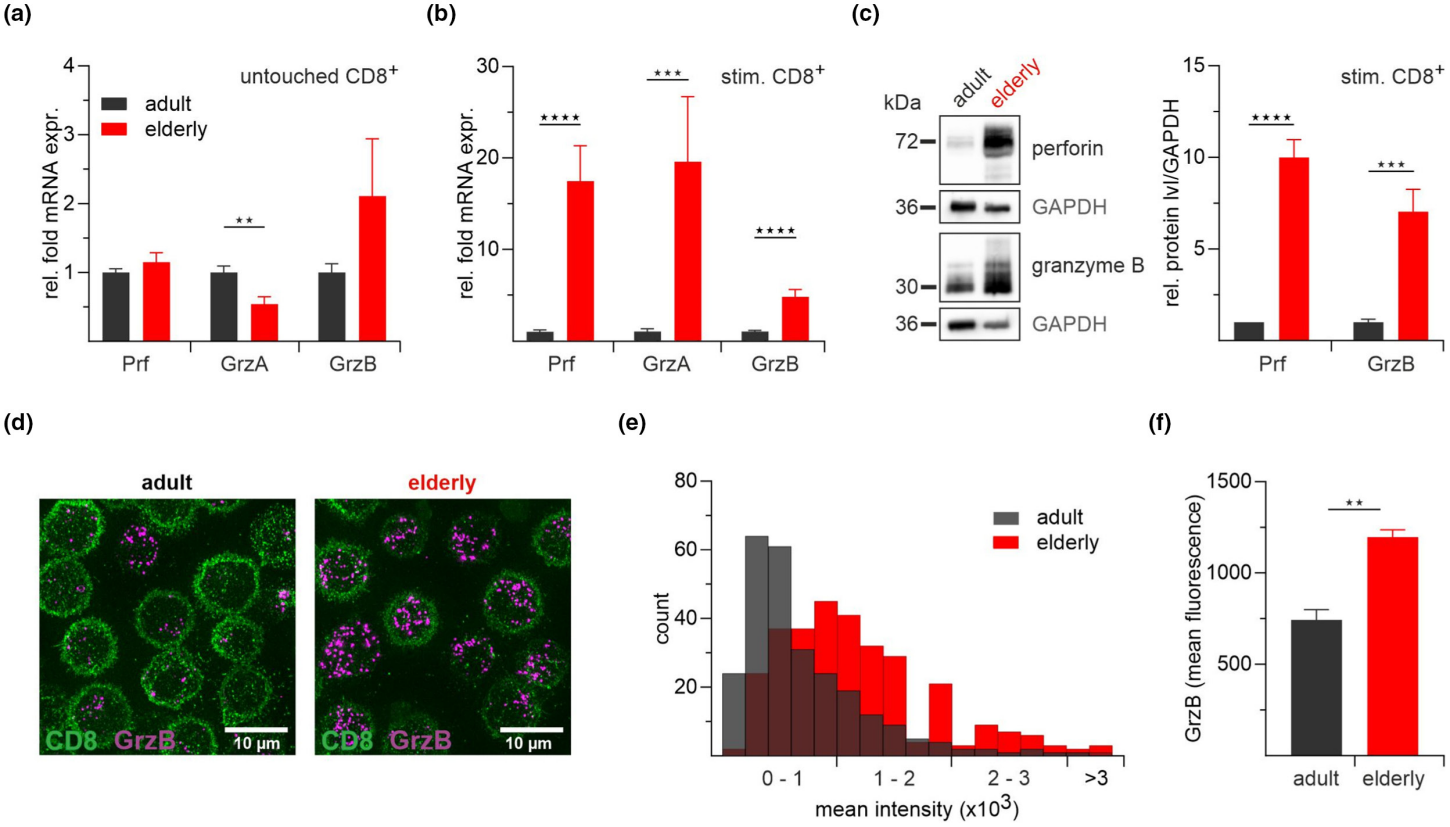
Increased perforin and granzyme expression in CD8^+^ T cells from elderly mice. Normalized mRNA expression of perforin (Prf) and the granzymes A (GrzA) and B (GrzB) in untouched (a) and stimulated (b) CD8^+^ T cells from adult and elderly mice. Expression levels were normalized to the reference genes hypoxanthine‐phosphoribosyl transferase 1 (HPRT1) and TATA box binding protein (TBP). Data from elderly mice (*n* = 7–8) are presented as relative fold change to the mRNA levels from adult mice (*n* = 8–13). (c) Perforin and granzyme B protein expression in stimulated CD8^+^ T cells from adult and elderly mice. Representative western blot and densitometric quantification with GAPDH as reference protein (*n* = 5–6). (d) Double immunofluorescence staining for granzyme B and CD8 in stimulated CD8^+^ T cells from adult and elderly mice. Representative SIM (Structured Illumination Microscopy) images after staining with Alexa647 anti‐human/mouse granzyme B and FITC anti‐mouse CD8a antibody. (e) Histogram of GrzB mean fluorescence intensity (bin width 200) and statistical analysis (f) of stained CD8^+^ T cells. Data are presented as mean ± SEM, *n* = 5–7

First, we performed qRT‐PCR and western blot experiments to investigate proteins relevant for granule exocytosis pathway, namely perforin and two major granzymes A and B. We found that, in untouched CD8^+^ T cells, mRNA expression levels of perforin, granzyme A and B were significantly reduced compared to the stimulated cells (Figure 6a,b). The mRNA expression levels of genes from elderly mice were normalized to reference genes and shown as relative fold change to the adult group. The untouched CD8^+^ T cells from elderly mice showed comparable mRNA transcript levels of perforin with a significant decrease in granzyme A expression (Figure 6a). In contrast to the untouched CD8^+^ T cells, the stimulated CD8^+^ T cells show a substantial increase in the mRNA transcript levels and protein levels for all three genes of interest (Figure 6b). Stimulated CD8^+^ T cells of elderly mice show a significant 17‐ to 19‐fold increase in perforin and granzyme A mRNA, and a 5‐fold increase in granzyme B compared to cells of the adult mice. We confirmed the observed mRNA increase by statistical quantification of the western blots after densitometry analysis (Figure 6c). Since SIM experiments show significantly more granzyme B^+^ granules per cell overall in CD8^+^ T cells from elderly mice (Figure 5a,b), we were interested if the granzyme B expression is evenly distributed throughout the CD8^+^ T cells in different age groups. Therefore, we fixed and stained the cells with anti‐granzyme B antibody coupled to Alexa647 and determined the mean fluorescence intensity (MFI) in both aged groups (Figure 6d–f). The analysis revealed a different distribution of granzyme B within T cells, with many cells having a very low MFI in the adult CD8^+^ T cells (Figure 6e). In contrast, the elderly mice's CD8^+^ T cells showed an increased number of cells with higher MFI (Figure 6e). The overall granzyme B intensity was increased by 45% in the elderly population (Figure 6f).

In addition to CTL's perforin and granzyme pathways, additional surface ligands and receptors can lead to altered killing kinetics. Increased expression of FasL has already been reported in old age (Aggarwal & Gupta, 1998). Besides FasL, NKG2D receptors are also relevant, particularly during the activation and immune response following infections and tumor growth (Verneris et al., 2004). Quantitative PCR assay detected significantly higher mRNA expression of FasL (Figure S5a) but no changes in NKG2D (Figure S5c) in CD8^+^ T cells of the elderly mouse compared to the adult. Although the expression of Fas on P815 cell is limited (Saxena & Adler, 1999), the receptor‐mediated pathway may contribute or support the faster killing kinetics in CD8^+^ T cells from the elderly. We directly assessed the contribution of Fas–Fas ligand interactions and the influence of NKG2D on the CD8^+^ T cell‐mediated cytotoxicity by treating cells with neutralizing antibodies against FasL (10 and 20 μg/ml) and NKG2D (10 μg/ml). We did not observe any effect (Figure S5b,d) and thus conclude that receptor‐mediated pathways play probably only minor roles or are masked by the dominant perforin/granzyme pathway.

Taken together, our data favor a model in which increased perforin and granzyme levels boost the speed of cancer cell elimination by increasing cytotoxic efficiency of individual CD8^+^ T cells with increasing age.

## 3 DISCUSSION

It is well established that, in elderly humans, pathogen clearance is impaired. This reduction is, among others, attributed to factors such as reduced proliferation of T cells including CD8^+^ cells and a reduced TCR repertoire (Goronzy & Weyand, 2017; Nikolich‐Zugich, 2018; Nikolich‐Zugich et al., 2012).

There are many CD8^+^ T cell studies that illustrate this reduced immunity. It has been shown in rodents that microbial pathogens known to cause substantial morbidity and mortality in elderly humans revealed significant CD8^+^ T cell defects (Nikolich‐Zugich, 2008; Nikolich‐Zugich et al., 2012). Especially infection of elderly organisms exhibited decreased numbers of effector CD8^+^ T cells with decreased expression of effector molecules leading to decreased cytolytic activity (Smithey et al., 2011). These functional changes may be the consequence of alterations intrinsic to the aged T cells themselves and/or extrinsic. The current data have revealed a variety of intrinsic and extrinsic factors that could influence the function of CD8^+^ T cells (Decman et al., 2010; Jergovic et al., 2019; Jiang et al., 2013; Lages et al., 2008). However, the exact mechanisms of these alterations are often not fully understood.

While it is commonly assumed that reduced cytotoxicity of CD8^+^ T cells is an important factor for reduced immunity, in vivo experiments provided evidence that clonal expansion and not cytotoxic activity of CD8^+^ T cells is the decisive factor of the observed reduced CTL response (Smithey et al., 2011).

It is critical to understand the genesis of the observed impairments, especially the efficacy of antiviral CD8^+^ T cells responses (Smithey et al., 2011; Yager et al., 2008), for the improvement of old and the development of new strategies to boost the immunity in elderly populations. To quantify cytotoxic capacity of CTLs is one critical factor to achieve this goal. We have used a real time kinetic assay to quantify cytotoxicity, which allows a much more detailed analysis than simply measuring an endpoint after a certain number of hours. The quantification of CTL killing kinetics against their targets revealed that CTLs from aged animals were much more efficient than their counterparts from adult mice. This result would have been easily overlooked by a simple endpoint analysis.

The results of endpoint lysis showed no significant difference in the cytotoxic ability between adult and elderly except an increased adult CD8^+^ T cell cytotoxicity for P815 as target cells. However, the endpoint lysis results depend very strongly on the target‐to‐killer cell ratio and the type of target cells itself. Similar observations regarding endpoint lysis were observed in previous studies conducted with entire splenocyte populations (Saxena & Adler, 1999; Saxena et al., 1988). Despite the similar endpoint lysis, we were able to show for the first time differences in the killing kinetics of murine CTLs against different tumor cell lines. Surprisingly, CD8^+^ T cells from elderly mice showed much steeper cytotoxicity kinetics during the first 60 min of target lysis. This is not only the case for the total CD8^+^ population, but also for the cytotoxic activity of the T_CM_ and T_EM_ in contrast to their young counterparts. Thus, CTLs (and in particular T_CM_ and T_EM_) of elderly mice display a much more efficient cytotoxicity against their targets than CTLs from adulty mice.

A detailed analysis of single CTLs revealed that perforin and granzyme B levels were higher in CTLs of elderly mice and that numbers of cells with successful cytotoxic granule fusion were significantly increased. The performed cytotoxic assay is predestined for observing fast lysis and consequently more focused on the fast‐acting exocytosis‐based pathway. These data support the conclusion that the perforin/granzyme pathway dominates the fast kinetics of CD8^+^ T cell cytotoxicity from elderly mice, in line with previous findings that the early phase of cytotoxicity analyzed by the real time killing assay is dominated by perforin/granzyme release (Backes et al., 2018; Zhou et al., 2018). However, the involvement of the slow‐acting ligand‐based pathway cannot be excluded entirely at this point and requires detailed investigation. One important question, which is difficult to resolve, is the mechanism how increased perforin/granzyme concentration in CTLs from elderly mice is achieved.

Upon TCR activation, transcription of perforin and granzyme genes is dynamically regulated by various signal transduction molecules, cytokines, and transcription factors involved in the Janus Kinase/Signal Transducer and Activator of Transcription (JAK/STAT) and Ca^2+^/Calcineurin/Nuclear Factor of Activated T cells (NFAT) signaling pathway. A well‐balanced interplay between activating signaling events and negative feedback regulation is crucial for maintaining T cell homeostasis and consequently the cytotoxic capacity of CTLs. Age‐related changes in TCR‐threshold calibration and altered expression of TCR costimulatory receptors, STAT‐dependent cytokines, and their negative regulators, suppressors of cytokines (SOCS), have already been described and could, at least partly, explain transcriptional modifications in CTLs from elderly mice (Fulop et al., 2014; Goronzy et al., 2012). However, to what extent single alterations decisively cause the increased perforin/granzyme expression, or if complex interactions or even compensatory mechanisms form these effective killer cells during aging, remains highly speculative. Moreover, we can only assume how much the faster cytotoxicity of CTLs may influence pathogen or cancer cell elimination in the inflammatory environment of an aged host. The very efficient cytotoxicity could counterbalance the many other reported deficits of immunity at an older age (Goronzy & Weyand, 2017; Nikolich‐Zugich, 2018; Nikolich‐Zugich et al., 2012). In this way, limited numbers of CTLs may still clear pathogens or fight cancer despite an overall reduced immunity. The use of efficient cytotoxic active CTLs in adoptive immunotherapy may boost their efficacy and extend lifespan. On the other hand, mounting a strong cytotoxic immune response may also come with disadvantages of an immune reaction which cannot be controlled well enough to be shut off at the right time. This may lead, especially in vivo, to highly increased cytokine concentration with potential dangers for the organism by triggering inflammatory response causing tissue damage and leading to pathogenesis of diseases (Small et al., 2001; Williams et al., 2021).

In summary, our findings reveal a yet undescribed altered phenotype of both CD8^+^ memory T cell subpopulations isolated from elderly mice. Elevated levels of perforin and granzymes result in faster cytotoxic activity transform the CTLs from elderly mice into ultimate killers. The underlying alteration appears to be intrinsic. Elucidating the molecular mechanisms may thus provide important targets for restoring/maintaining immune balance during aging.

## 4 EXPERIMENTAL PROCEDURES

### 4.1 Mice

*C57BL6/J* mice were purchased from Charles River Laboratories and bred in our own colonies. All animal experiments were approved by local authorities and were performed in compliance with the German Animal Protection Law (Tierschutzgesetz, §11, Abs.1 Nr.1). Mice were housed under specific‐pathogen‐free conditions and sacrificed by cervical dislocation at the designated time. Only female mice between 12 and 24 weeks (adult mice) and 78 and 102 weeks (elderly mice) were used for this study. Mice with splenomegaly (spleen to body weight ratio above 0.6) or macroscopically visible tumors were excluded.

Spleens were harvested and splenocytes were isolated using a 40 μm cell strainer (Corning®). Erythrocytes were lysed by incubation with a hypoosmolar solution. CD8^+^ T cells were negatively isolated using the Dynabeads™ Untouched™ Mouse CD8 Cells Kit (ThermoFisher). Naïve CD8^+^ T cells were isolated with the Naive CD8a^+^ T Cell Isolation Kit (Miltenyi). The purity and subtype distribution were evaluated by flow cytometry.

### 4.2 Cell culture

Murine CD8^+^ T cells were cultured in AIM V medium (Thermo Fisher Scientific), supplemented with 10% FCS, 50 μM ß‐mercaptoethanol and 100 U/ml recombinant human IL‐2 (Miltenyi), if not stated otherwise. For stimulation, Dynabeads Mouse T‐Activator CD3/CD28 for T cell Expansion and Activation (Thermo Fischer Scientific) were added with a 5:4 cell‐to‐bead ratio. Stimulated CD8^+^ T cells were maintained at 37°C and 5% CO_2_ for up to 6 days. P815 cells (ATCC, #TIB‐64) and EL4 cells (ATCC, #TIB‐39) were cultured in RPMI‐1640 (Thermo Fisher Scientific), supplemented with 10% FCS and 1% penicillin/streptomycin.

### 4.3 Real time killing assay

Real‐time killing assays were carried out as described previously (Kummerow et al., 2014). P815 and EL4 target cells were loaded with 500 nM calcein‐AM in AIM V medium containing 10 mM HEPES for 15 min at room temperature. Cells were washed once and settled into black 96‐well plates with clear‐bottom (Corning®) at a density of 2.5 × 10^4^ cells/well. CD8^+^ T cells were pulsed with 2 μg/ml anti‐CD3ε antibody (Biolegend) and gently added onto target cells at the indicated effector‐to‐target ratio. To block NKG2D‐ and FasL‐mediated cytotoxicity, CD8^+^ T cells were treated with 10 and 20 μg/ml neutralizing antibodies (Biolegend). Target cell lysis was measured with a GENios Pro plate reader (Tecan) every 10 min for 4 h at 37°C using bottom reading mode and analyzed as described before (Kummerow et al., 2014).

### 4.4 Live cell imaging

P815 target cells were loaded with 500 nM calcein‐AM and settled into black 96‐well plates with clear bottom as described above. To counterstain necrotic target cells, 400 ng/ml propidium iodide (PI) was added to each well. CD8^+^ T cells were pulsed with 2 μg/ml anti‐CD3ε antibody (Biolegend) and gently added onto target cells at a 2:1 effector‐to‐target ratio. Image acquisition was done with the high‐content imaging system ImageXpress Micro XLS (Molecular Devices), which is equipped for 37°C and 5% CO_2_ incubation. For excitation, the Spectra X LED illumination (Lumencor) was used. Calcein was excited with the LED 470/24 nm with 10% intensity for 5 ms and PI with the LED 542/27 nm with 10% intensity for 10 ms. The emission filter settings 520/35 nm (Calcein) and 692/40 nm (PI) were used. A 20 x S Fluor 0.75 numerical aperture objective (Nikon) was used. 2 sites per well were imaged every 2 min for 2 h. For creating composites and for further analysis ImageJ was used. Therefore, 50 cells per site were randomly chosen and analyzed for time point of contact with a CD8^+^ T cell, the loss of calcein fluorescence, and the gain of PI fluorescence. Cells without any contact with a killer cell or cells moving out of the imaged site were not further analyzed.

### 4.5 Flow cytometry and cell sorting

All antibodies used for flow cytometry were purchased from Biolegend. For cell sorting, mouse splenocytes or stimulated CD8^+^ T cells were stained with FITC‐conjugated anti‐CD8, PE‐conjugated anti‐CD44 and APC‐conjugated anti‐CD62L antibody, incubated for 20 min at room temperature and washed once in PBS + 0.5% BSA. Cell sorting was performed on a FACSAria™III (BD Biosciences) with 85 μM nozzle. To evaluate purity and subtype distribution of sorted or isolated CD8^+^ T cells, at least 2.5 × 10^5^ cells were stained with PerCP‐conjugated anti‐CD3, Pacific Blue‐conjugated anti‐CD4, FITC‐conjugated anti‐CD8, PE‐conjugated anti‐CD44 and APC‐conjugated anti‐CD62L antibody. 2x10^4^ cells per sample were acquired on a BD FACSVerse™ flow cytometer (BD Biosciences) and analyzed using FlowJo version 10 (FlowJo, LLC).

### 4.6 Proliferation assay

Untouched CD8^+^ T cells were labeled with CFSE using the CellTrace CFSE Cell Proliferation Kit (Thermo Fischer Scientific) and stimulated for 48 h with 100 U/ml recombinant human IL‐2 (Miltenyi) and Dynabeads Mouse T‐Activator CD3/CD28 for T cell Expansion and Activation (Thermo Fischer Scientific). Stimulated cells were stained with PerCP‐conjugated anti‐CD8 antibody, and cell division was quantified by flow cytometry.

### 4.7 Degranulation assay

Stimulated CD8^+^ T cells were pulsed with 2 μg/ml anti‐CD3ε antibody (Biolegend) and incubated with 3 × 10^5^ P815 cells (E:T 1:1) in AIM V medium for the indicated time points at 37°C and 5% CO_2_ in the presence of Brilliant violet 421‐conjugated anti‐CD107a antibody (Biolegend). Cells were washed once in cold PBS + 0.5% BSA and stained with FITC‐conjugated anti‐CD8, PerCP‐conjugated anti‐CD44 and APC‐conjugated anti‐CD62L antibody (Biolegend). 3 × 10^4^ cells per sample were analyzed by a BD FACSVerse™ Flow Cytometer (BD Biosciences). Median fluorescence intensity (MFI) of CD107a was determined in CD8^+^CD44^+^CD62L^+^ (T_CM_) and CD8^+^CD44^+^CD62L^−^ (T_EM_) cells using FlowJo software version 10 (FlowJo, LLC).

### 4.8 Total internal reflection fluorescence microscopy (TIRF)

Stimulated CD8^+^ T cells from adult and elderly mice were electroporated (Mouse T cell Nucleofector kit, Lonza) with pMAX‐GranzymeB‐mcherry to mark the cytotoxic granules. After 14 h of transfection, 0.2 × 10^6^ cells were washed and suspended in 40 μl extracellular solution (2 mM Hepes, 140 mM NaCl, 4.5 mM KCl, and 2 mM MgCl_2_) containing no Ca^2+^ and settled onto anti‐CD3ε antibody (30 μg/ml)‐coated coverslips. Cells were then perfused with extracellular buffer containing 10 mM Ca^2+^ to visualize lytic granule fusion at the TIRF plane using the 100× Plan‐Apochromat objective (NA 1.45). The acquisition frequency was 10 Hz, and the exposure time was 100 ms. The TIRFM setup was from Olympus (Olympus Europa SE and Co KG) and equipped with a solid‐state laser 85 YCA emitting at 561 nm (Melles‐Griot). Images were captured with a QuantEM512SC camera (Photometrics) and was controlled by VisiView software (Version:4.0.0.11, Visitron GmbH). Fusion of granules was analyzed using Fiji v1.46. A sudden drop in granzyme B‐mcherry fluorescence within 3 frames (0.3 s) was defined as fusion event (Ming et al., 2015).

### 4.9 Quantitative real‐time PCR

Total RNA from stimulated CD8^+^ T cells was isolated using TRIzol® Reagent (Thermo Fisher Scientific). 0.8 μg total RNA was reverse transcribed, and 1 μl of cDNA was used for realtime PCR. Quantitative real‐time PCR assays were conducted in a CFX96™ Real‐Time System C1000™ Thermal Cycler (BioRad) using the QuantiTect SYBR Green PCR Kit according to the manufacturer's instructions (Qiagen). Expression of target genes were normalized to the expression of the reference genes HPRT1 and TBP. Relative expression levels were calculated using the ΔCq method (2^−ΔCq^).

QuantiTect primers:

**Table:** 

Target gene	Product	Cat. no.
Perforin	Mm_Prf1_1_SG	QT00282002
Granzyme A	Mm_Gzma_1_SG	QT00100667
Granzyme B	Mm_Gzmb_1_SG	QT00114590
FasL	Mm_Tnfsf6_1_SG	QT00104125
NKG2D	Mm_Klrk1_1_SG	QT00104363
HPRT1	Mm_Hprt_1_SG	QT00166768
TBP	Mm_Tbp_1_SG	QT00198443

### 4.10 Western blot analysis

Stimulated CD8^+^ T cells were lysed in 1× RIPA buffer and proteins extracted by sonication.

Protein concentration was determined using the Pierce™ BCA™ Protein Assay Kit (Thermo Fisher). Equivalent amounts of proteins were denatured in Laemmli buffer under reducing conditions, separated by 7%–10% SDS‐PAGE and transferred to PVDF‐membrane using a transblot electrophoresis transfer cell (Fisherbrand). Membranes were blocked with 5% skim milk powder in Tris–HCl‐buffered saline/0.1% Tween (TBST) for 1 h, incubated overnight with primary antibodies against perforin (Invitrogen), granzyme B (Cell Signaling), and GAPDH (Cell Signaling), washed in TBST and incubated with horseradish peroxidase‐coupled secondary antibodies (GE Healthcare) for 1 h. For protein detection, an enhanced chemiluminescence detection reagent (BioRad) was used. Densitometric quantification was done with Quantity one software (Bio‐Rad).

### 4.11 Immunocytochemistry (ICC) and Structured illumination microscopy (SIM)

Stimulated CD8^+^ T cells (0.4 × 10^6^ cells) from adult and elderly mice were stained with anti‐granzyme B antibody (Biolegend) or anti‐CD8a antibody (BD). Briefly, CD8^+^ T cells were washed with PBS and for cell surface staining, FITC anti‐mouse CD8a antibody (1:100) was used and stained on ice to prevent endocytosis. The cells were washed once and settled onto 0.01% poly‐ornithine coated coverslips. The cells were then fixed with ice‐cold 4% PFA solution, washed 3 times with PBS and permeabilized with 0.1% TritonX‐100 (Sigma), then blocked with blocking buffer (0.1% TritonX and 5% BSA in PBS). The cells were then stained with anti‐human/mouse granzyme B coupled to Alexa647 (1:200) for 45 mins and mounted for SIM analysis (Zeiss ELYRA PS.1; Carl Zeiss Microscopy GmbH). Images were acquired by using Zen2012 software and a 63× Plan‐Apochromat (NA 1.4) objective was used with laser excitation of 488 and 561 nm. The images were then processed using the same software to obtain higher resolution. Z‐stacks of 200‐nm step size were used to scan the cells. The images were then analyzed using Fiji v1.46, and the granule count was done using 3D objects counter plugin.

### 4.12 Statistical analysis

Data are presented as mean ± SEM (*n* = number of experiments) if not stated otherwise. Data were analyzed using GraphPad (Prism) software version 8 and Microsoft Excel 2016. If Gaussian distribution was confirmed, unpaired Student's *t* tests were performed to evaluate statistical significance. If no Gaussian distribution was given, nonparametric Mann–Whitney tests were performed. Degrees of significance were set at *p < 0.05, **p < 0.01, ***p < 0.001 and ****p < 0.0001.

## AUTHOR CONTRIBUTIONS

A.L. and D.Z. designed the study. A.L., D.Z. and M.H. discussed and interpreted all data, and wrote the manuscript with input from all authors. D.Z., A.A. and C.H. isolated murine CD8^+^ T cells, performed real‐time killing assays, flow cytometry analysis and degranulation assay. L.K. performed single target cell death experiments and analysis. K.R. did all SIM and TIRF experiments and analysis. S.J. performed qPCR and western blot experiments. D.Z. performed statistical analysis and designed final figure layout.

## CONFLICT OF INTEREST

Authors declare that they have no competing interests.

## Supporting information


FIGURES S1–S5

## Data Availability

The data that support the findings of this study are available from the corresponding author upon reasonable request.
