# Supplementary material for: Faster cytotoxicity with age: Increased perforin and granzyme levels in cytotoxic CD8 + T cells boost cancer cell elimination
Source: Aging Cell. 2022 Jul 11;21(8):e13668. doi: 10.1111/acel.13668 (PMC9381916; doi:10.1111/acel.13668)
Supplement: Supplementary file 1 — FIGURES S1–S5 [file ACEL-21-e13668-s001.pdf]

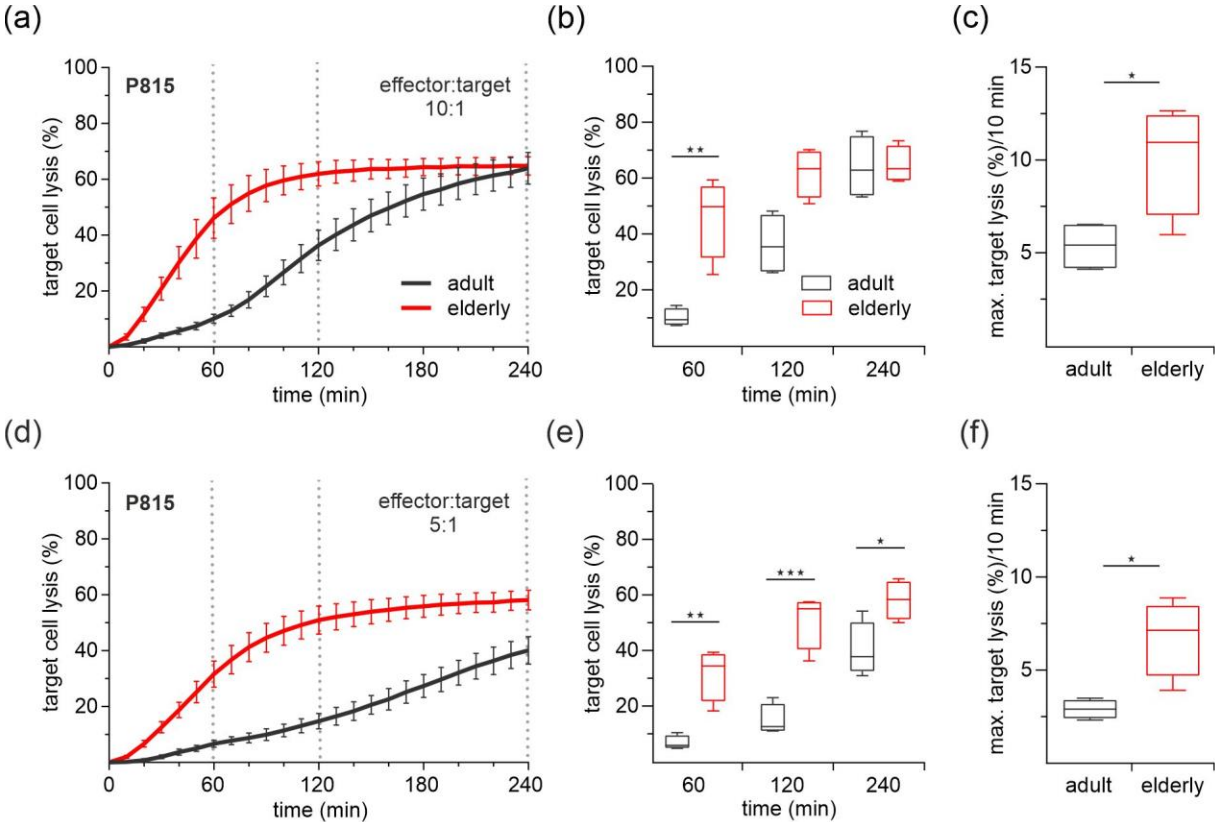

FIGURE S1 Differences in killing kinetics between $CD8^+$ T cells from adult and elderly mice are independent of effector-to-target cell ratios. Time-resolved killing assays with stimulated $CD8^+$ T cells from adult and elderly mice. P815 cells were used as target cells in an effector-to-target ratio of 10:1 (a) and 5:1 (d). Box plots represent the average target cell lysis after 60, 120, and 240 min (b, e) and the maximum target lysis per 10 min (c, f). Data are presented as mean ± S.E.M, n=4.

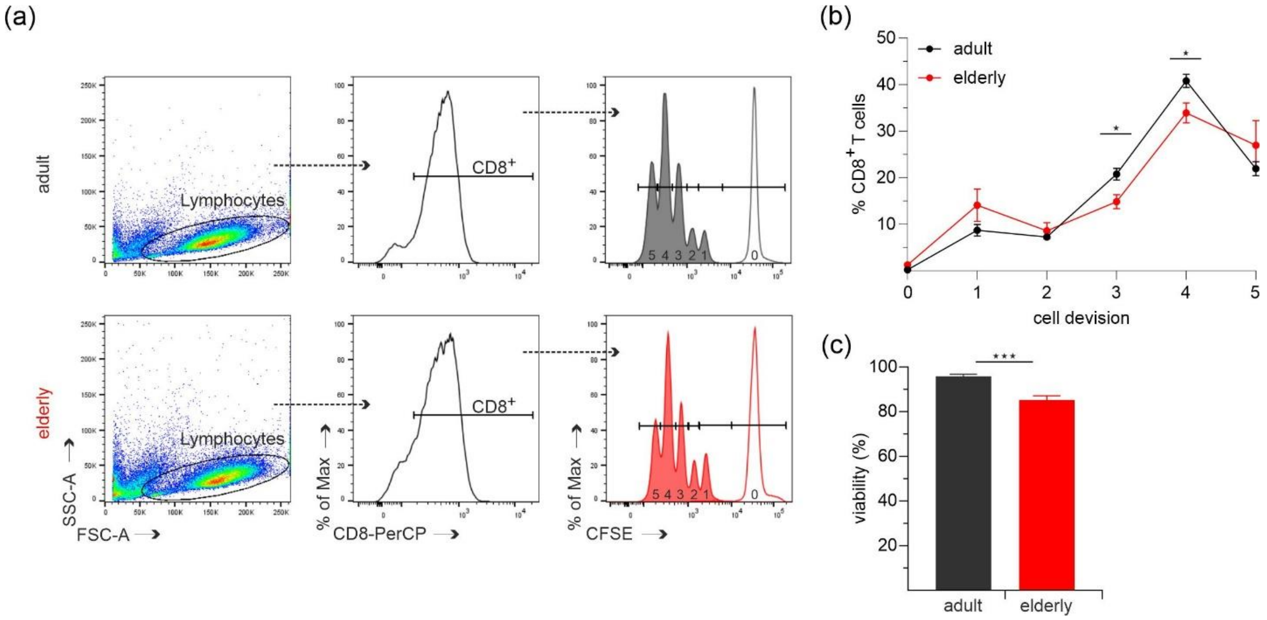

FIGURE S2 Decreased proliferation and viability of CD8^{+} T cells from elderly mice. Flow cytometry-based proliferation assay with stimulated CD8^{+} T cells from adult (black) and elderly (red) mice. (a) Representative gating strategy and (b) quantification of cell division by dilution of cytoplasmatic dye CFSE 48h after stimulation (n=6). (c) Viability of 3-day stimulated CD8^{+} T cells was analyzed by staining the cells with Acridine Orange/Propidium Iodide and using an automated fluorescence cell counter (n=8). Data are presented as mean ± S.E.M.

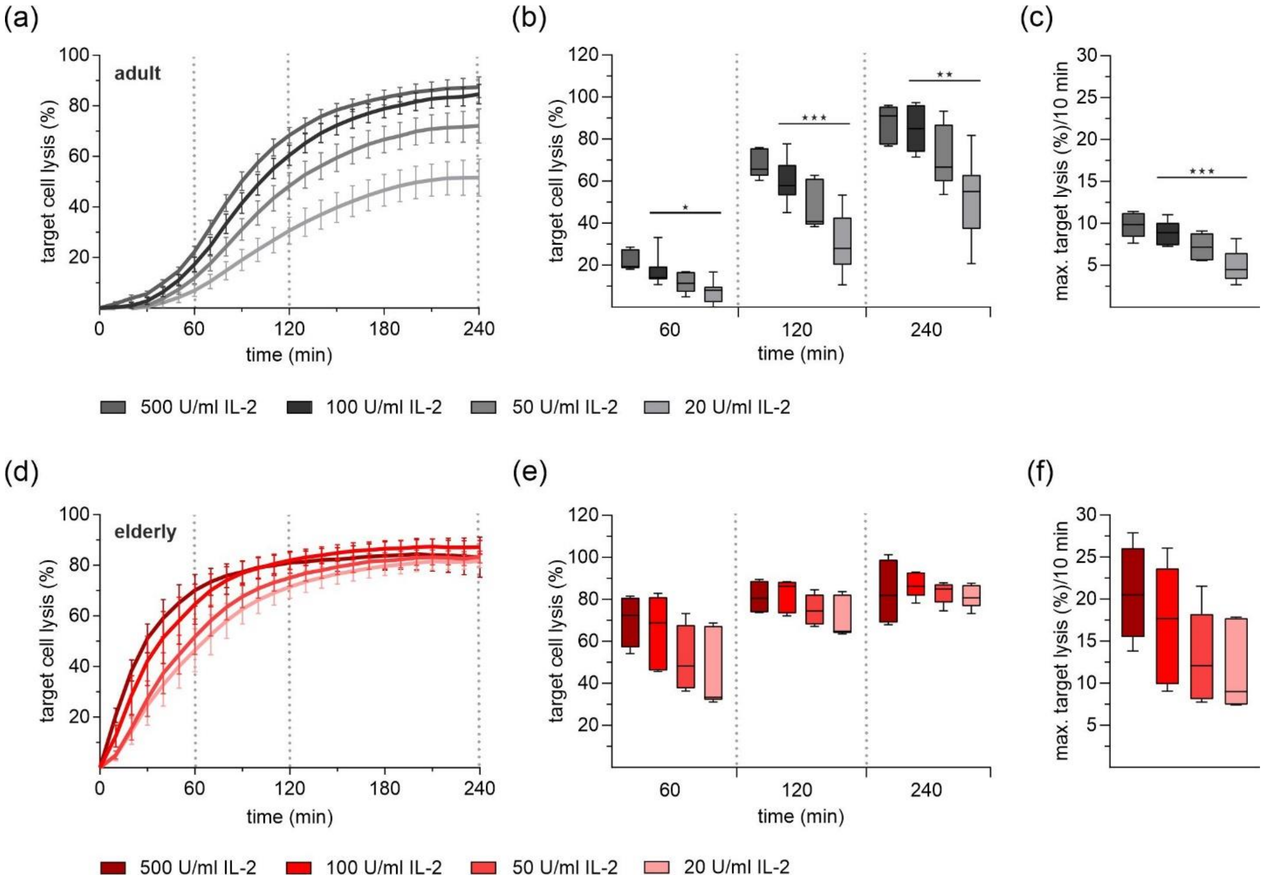

FIGURE S3 Killing kinetics of CD8$^+$ T cells from elderly mice remains unaffected under low IL-2 conditions. Time-resolved killing assays with CD8$^+$ T cells from adult (a) (n=5-7) and elderly (d) (n=4-5) mice after three days of stimulation with varying IL-2 concentrations. P815 cells were used as target cells in an effector-to-target ratio of 20:1. Box plots represent the average target cell lysis after 60, 120, and 240 min (b, e) and the maximum target lysis per 10 min (c, f) as a measure of the kinetics. Data are presented as mean ± S.E.M.

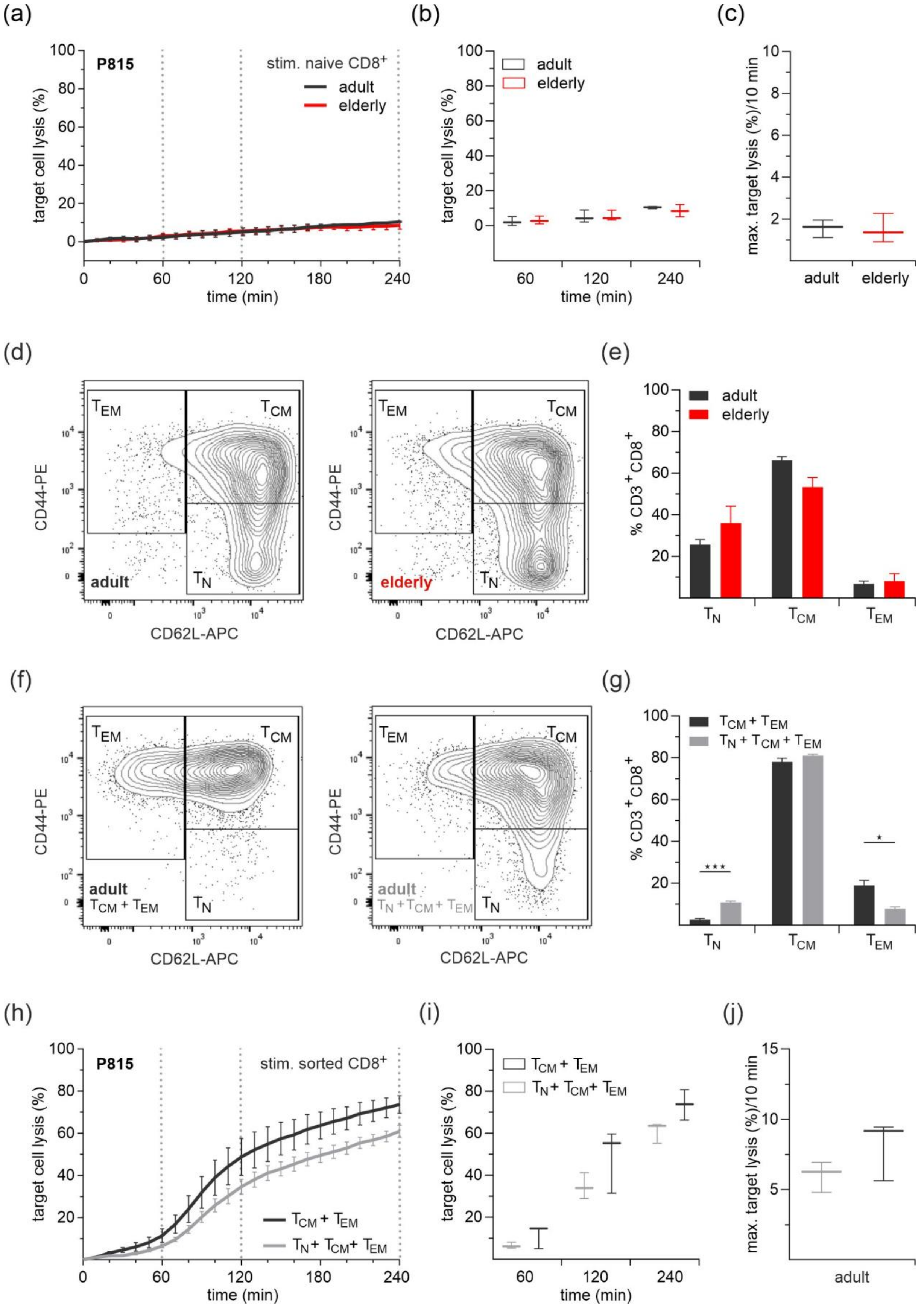

(a)
P815
stim. naive CD8+
adult
elderly
target cell lysis (%)
time (min)
(b)
adult
elderly
target cell lysis (%)
time (min)
(c)
max. target lysis (%)/10 min
adult
elderly
(d)
TEM
TCM
TN
CD44-PE
CD62L-APC
adult
elderly
(e)
adult
elderly
% CD3+CD8+
TN
TCM
TEM
(f)
TEM
TCM
TN
CD44-PE
CD62L-APC
adult
TCM + TEM
adult
TN + TCM + TEM
(g)
TCM + TEM
TN + TCM + TEM
% CD3+CD8+
***
*
TN
TCM
TEM
(h)
P815
stim. sorted CD8+
TCM + TEM
TN + TCM + TEM
target cell lysis (%)
time (min)
(i)
TCM + TEM
TN + TCM + TEM
target cell lysis (%)
time (min)
(j)
max. target lysis (%)/10 min
adult

FIGURE S4 The proportion of naïve $CD8^+$ T cells does not determine killing kinetics. Time-resolved killing assays with naïve $CD8^+$ T cells isolated from adult and elderly mice after three days of stimulation and P815 as target cells (a). Box plots represent the average target cell lysis after 60, 120, and 240 min (b) and the maximum target lysis per 10 min (c) Representative contour plots (d) and analysis (e) of subtype distribution after stimulation of naïve $CD8^+$ T cells from adult and elderly mice. Sorted T_{CM} + T_{EM} from adult mice were stimulated without or together with sorted naïve T cells in a 60 to 40% ratio. After three days of stimulation, analysis of $CD8^+$ subtype distribution (f, g) and time-resolved killing assays (h, i, j) were performed. Data are presented as mean ± S.E.M., n=3.

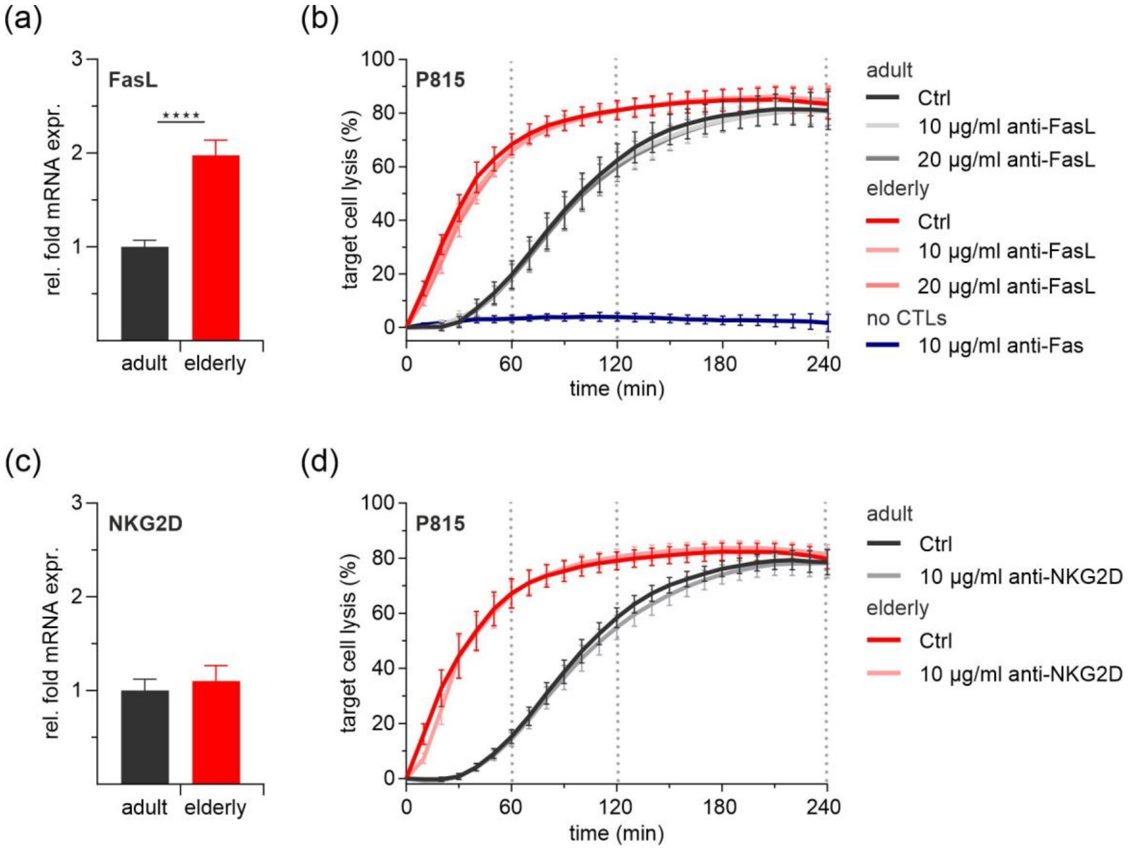

FIGURE S5 FasL- and NKG2D-mediated cytotoxicity do not change faster killing kinetics of $CD8^+$ T cells from elderly mice. Normalized mRNA expression of FasL (a) and NKG2D (c) in stimulated $CD8^+$ T cells from adult (black) and elderly (red) mice. Expression levels were normalized to the reference genes hypoxanthine-phosphoribosyl transferase 1 (HPRT1) and TATA box binding protein (TBP). Data from elderly mice (n=4-6) are presented as relative fold change to the mRNA levels from adult mice (n=7-8). Time-resolved killing assays were performed after adding an activating anti-Fas antibody to P815 cells (without CTLs) or treating stimulated $CD8^+$ T cells with neutralizing antibodies to FasL (**b**) and NKG2D (**d**). P815 cells were used as target cells in an effector-to-target ratio of 20:1 (n=4-5). Data are presented as mean ± S.E.M
